# Rapid analysis of right ventricular volumes and systolic function using Cardiovascular Magnetic Resonance Imaging

**DOI:** 10.1186/1532-429X-11-S1-P254

**Published:** 2009-01-28

**Authors:** Simon C Koestner, Marco JW Götte, Robin Nijveldt, Jan GJ Groothuis, Aernout M Beek, Mark BM Hofman, Albert C van Rossum

**Affiliations:** VU University Medical Center, Amsterdam, Netherlands

**Keywords:** Cardiovascular Magnetic Resonance, Right Ventricle, Cardiovascular Magnetic Resonance Image, Right Ventricle Function, Cine Cardiovascular Magnetic Resonance

## Introduction

The function of the right ventricle (RV) is an important prognostic factor in heart disease. Assessment of RV function using the gold standard disk-area (or Simpson) technique is time consuming and semi-quantitative evaluation (e.g. TAPSE) shows unsatisfactory correlation with quantitative analysis. The assessment of left ventricular function with a biplane area-length method has been validated for invasive ventriculography and for CMR. This method has never been used for the RV because of its particular geometry.

## Purpose

This study investigates the use of an area-length technique for the rapid assessment of the volumes and function of the RV using cardiovascular magnetic resonance imaging (CMR).

## Methods

12 healthy volunteers and 25 patients with RV dilation and/or dysfunction (9 with pulmonary hypertension (PHT), 9 with dilated RV without PHT, 7 with ischemic heart disease) underwent cine CMR on a 1.5 T scanner. RV end-diastolic volume (RVEDV), end-systolic volume (RVESV) and ejection fraction (RVEF) were calculated using a stack of short-axis cines as the standard of reference (Simpson technique). A 4-chamber view and a perpendicular RV 2-chamber view were acquired to calculate RV volumes and RVEF using a simple area-length method (A*A/L) without correction coefficient in both end-diastolic and end-systolic phases with A the RV area, and L the apex to base length. This was tested for both a single and a biplane long-axis view. Finally, TAPSE was measured on the 4-chamber view. Area-length methods and TAPSE were compared to the standard of reference.

## Results

Analysis of the area-length method using long-axis orientations was faster (~2 min) than the Simpson method (~20 min). The estimation of RVEF using biplane area-length method correlated well with short-axis calculation (r^2^_2LA_ = 0.72, p < 0.01). However, this method slightly overestimated RVEF (difference 5.5%, p = 0.045). The correlation between RVEF and single long-axis analysis (4-chamber) or TAPSE was weaker (r^2^_1LA_ = 0.49, p < 0.01 and r^2^_TAPSE_ = 0.37, p < 0.01, respectively). There was a good correlation between the two orientations in RVEDV measurements (r^2^_2LA_ = 0.78, p < 0.01) and in ESV measurements (r^2^_2LA_ = 0.82, p < 0.01) using the biplane method. There was no significant volume differences between the two methods (EDV: mean_2LA_ = 196.5 ± 92.4 mL, mean_SIMPSON_ = 208.4 ± 78.7 mL, p = 0.55; ESV: mean_2LA_ = 104.2 ± 70 mL, mean_SIMPSON_ = 119.0 ± 58.4 mL, p = 0.33).

## Conclusion

Measurements of RV volumes and RVEF using biplane area-length method correlate well with the gold standard of contiguous short-axis measurements, despite the particular geometry of the RV. However, this method leads to a 5% overestimation of RVEF which should be taken in consideration in the RVEF calculation. As analysis time is significantly shorter, this method can be used as a rapid, quantitative screening tool.

